# Effects of Histamine Receptor Antagonist Cetirizine on Orthodontic Tooth Movement

**DOI:** 10.3390/biomedicines8120583

**Published:** 2020-12-08

**Authors:** Gregor Sperl, Johanna Gattner, James Deschner, Michael Wolf, Peter Proff, Agnes Schröder, Christian Kirschneck

**Affiliations:** 1 Department of Orthodontics, University Hospital Regensburg, 93053 Regensburg, Germany; gregor.sperl@stud.uni-regensburg.de (G.S.); johanna.gattner@stud.uni-regensburg.de (J.G.); peter.proff@ukr.de (P.P.); agnes.schroeder@ukr.de (A.S.); 2 Department of Periodontology and Operative Dentistry, University of Mainz, 55131 Mainz, Germany; james.deschner@uni-mainz.de; 3 Department of Orthodontics, University Hospital RWTH Aachen, 52062 Aachen, Germany; michwolf@ukaachen.de

**Keywords:** cetirizine, orthodontic tooth movement, histamine receptor antagonists, µCT

## Abstract

Many patients regularly take histamine receptor antagonists, such as cetirizine, to prevent allergic reactions, but these antiallergic drugs may have inadvertent effects on orthodontic treatment. In previous studies, histamine has been shown to modulate the sterile inflammatory reaction underlying orthodontic tooth movement. Pertinent effects of histamine antagonization via cetirizine during orthodontic treatment, however, have not been adequately investigated. We thus treated male Fischer344 rats either with tap water (control group) or cetirizine by daily oral gavage corresponding to the clinically used human dosage adjusted to the rat metabolism (0.87 mg/kg) or to a previously published high dosage of cetirizine (3 mg/kg). Experimental anterior movement of the first upper left molar was induced by insertion of a nickel-titanium (NiTi) coil spring (0.25 N) between the molar and the upper incisors. Cone-beam computed tomography (CBCT), micro-computed tomography (µCT) images, as well as histological hematoxylin-eosin (HE), and tartrate-resistant acid phosphatase (TRAP) stainings were used to assess the extent of tooth movement, cranial growth, periodontal bone loss, root resorptions, and osteoclast activity in the periodontal ligament. Both investigated cetirizine dosages had no impact on the weight gain of the animals and, thus, animal welfare. Neither the extent of tooth movement, nor cranial growth, nor root resorption, nor periodontal bone loss were significantly influenced by the cetirizine dosages investigated. We, thus, conclude that histamine receptor antagonist cetirizine can be used during orthodontic treatment to prevent allergic reactions without clinically relevant side effects on orthodontic tooth movement.

## 1. Introduction

Histamine, which is stored in mast cells and basophils [1], is an essential mediator in the pathogenesis of various allergic diseases, such as atopic dermatitis, rhinitis, and asthma [2], and plays a role in bone metabolism [3,4,5,6]. To exert its biological effects, histamine must bind to a histamine receptor. Four different histamine receptors (H1–H4) have been described, which are distributed throughout various tissues and cells of the human body [7]. H1-receptors (H1R) are located on blood vessels and sensory nerves and increase vascular permeability, stimulate sensory nerves of airways and promote chemotaxis of eosinophils [7]. The H1R is primarily responsible for the symptoms of rhinitis such as sneezing, nasal congestion, and rhinorrhea [7]. Cetirizine is a second-generation H1-antihistamine, which blocks histamine during type-I hypersensitivity reactions [8]. It is clinically used to treat allergic diseases, such as chronic urticaria and allergic rhinitis and freely available as over-the-counter medication [1]. Allergic diseases are common within European countries with a prevalence ranging from 1.4% to 31.6% and a median prevalence of 16.9% [9]. There is most likely a significant overlap between people who consume antihistamines, such as cetirizine and people who undergo orthodontic treatment. Orthodontic tooth movement (OTM) to correct misaligned teeth and malocclusions therapeutically occurs when prolonged force is applied to a tooth by removable or fixed orthodontic appliances. The surrounding alveolar bone responds to the applied force by selectively removing bone material in pressure areas and adding it in tension areas [10]. This response is mediated by the periodontal ligament (PDL) [10], which is mainly comprised by fibroblasts and collagenous fibers [11]. The PDL is a connective tissue located between the cementum covering the root of the tooth and the bone, forming the socket wall [12]. Besides its primary function to support the teeth in their sockets and allowing them to withstand the forces of mastication, the PDL acts as a sensory receptor for the positioning of the jaws during mastication and is a cell reservoir for tissue homeostasis and regeneration [12]. Osteoclasts remove bone from the area adjacent to the compressed part of the PDL, while osteoblasts form new bone on the tension side and are involved in bone remodeling and osteoclast activation on the pressure side [10]. The biological response of cells to OTM has been described as an aseptic inflammation, as it is mediated by inflammatory cytokines, but does not represent a pathological condition [13]. A variety of inflammatory factors, such as prostaglandins, interleukins, tumor necrosis factor, as well as the receptor activator of nuclear factor-κB ligand (RANKL) are produced by PDL fibroblasts, macrophages, and immune cells in response to OTM [13].

An in vitro study showed that histamine induced the release of prostaglandin E_2_ via H1 receptor-mediated Ca^2+^ signaling in PDL cells [14]. More recently published research demonstrated that human PDL fibroblasts express H1-, H2-, and H4-receptors [15]. In previous studies a wide variety of drugs, which can be diffusely consumed even without the medical prescription at the same time of an orthodontic treatment, have already been investigated regarding a potential influence on OTM [16]; however, to date little is known about the effects of cetirizine on OTM. Merely two previous studies investigated OTM in rats with application of cetirizine [17,18]. Unfortunately, the authors overdosed cetirizine in their studies. A more recent study investigated the effects of cetirizine on bone remodeling after calvarial suture expansion in rats [19]. Application of cetirizine increased bone formation in the expanded suture area and led to a narrower average suture width at day 28 [19]. In contrast, cetirizine did not affect bone mineral density or bone quality in a study investigating osteoporotic phenotype and wild type mice [4]. Another study investigated loratadine, which is a second generation H1-antihistamine-like cetirizine, and its effect on the skeletal system of rats [20]. A high dose of loratadine slightly, but significantly, affected the development of the skeletal system in rapidly growing rats [20]. 

Orthodontically induced inflammatory root resorption (OIIRR) is considered an unavoidable pathologic consequence of OTM [21], with severe OIIRR occurring in up to 2.9% of teeth treated with fixed orthodontic appliances [22]. Individual susceptibility, genetics, and systemic factors have been suggested as significant modulators of OIIRR [13]. Recent studies also investigated drugs and hormones as a potential influence on OIIRR [23,24,25,26,27], but to date, no research is available regarding cetirizine. Periodontal complications and subsequent alveolar bone loss are another possible side effect of orthodontic treatment, but can usually be controlled by proper oral hygiene [28,29,30]. To date no radiographic analysis of the periodontal bone after OTM and cetirizine administration has been performed.

Due to the prevalence of allergies mediated by the H1-receptor, the use of cetirizine generally increased in the population. Due to its systemic anti-inflammatory action, an impact on OTM is to be considered, as OTM is also a (sterile) inflammatory process at the molecular level mediated by periodontal ligament fibroblasts [31], which have been shown before to change their expression pattern under the influence of histamine [15]. This animal study, therefore, aims to further investigate the influence of the H1-receptor antagonist cetirizine on the rate of OTM, cranial growth, root resorption, and periodontal bone loss in an in vivo rat model of orthodontic tooth movement.

## 2. Materials and Methods

### 2.1. Experimental Animals and Housing

All animal experiments were performed according to German law (55.2.2-2532.2-826.24, 28 January 2019, government of Lower Franconia, Germany) in compliance with the ARRIVE guidelines (Animal Research: Reporting of In Vivo Experiments). Male inbred Fischer-344 rats were ordered from Charles River Laboratories (Sulzfeld, Germany). The animals had a mean body weight of 170 ± 17.5 g and were 7 weeks of age at the beginning of the medication. The Fischer-344 strain was already successfully used in previous research investigating orthodontic tooth movement in rats [25,27,32]. The animals were housed in a conventional animal laboratory in type IV metal grid polycarbonate cages under constant noise-free environmental conditions, 25 Pa overpressure, 16 air changes/h, 55 ± 10% humidity, 21 ± 1 °C room temperature and an automatic night-day cycle of 12:12 h with the light phase ranging from 7:00 a.m. to 7:00 p.m. A standard rat maintenance diet (V1535, ssniff-Spezialdiäten GmbH, Soest, Germany) and tap water were provided ad libitum. After insertion of the orthodontic appliance the food pellets were mixed to a mash to prevent mechanical deformation of the orthodontic appliance. The arrival of the animals in the laboratory was followed by a two-week acclimatization period, in which they got accustomed to handling and oral gavaging. 

### 2.2. Study Design, Sample Size, and Allocation 

**Figure 1:**
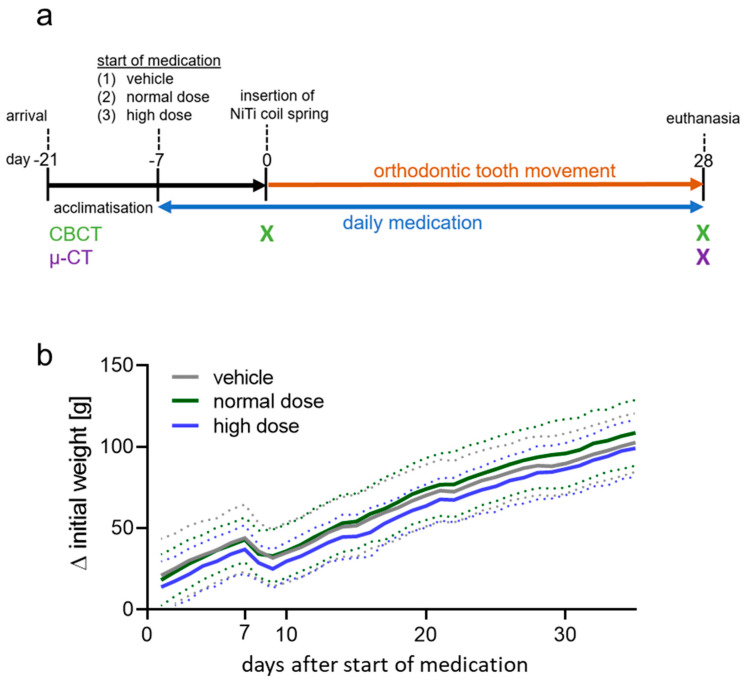
(**a**) Experimental time-flow chart of the animal experiment. (**b**) Weight difference to initial gross body weight of rats treated with tap water (vehicle), normal or high dose cetirizine. Solid line: mean values; dashed line: standard deviation. n ≥ 19. Statistics: Welch-corrected ANOVA followed by Games–Howell multiple comparison tests.

The rats were randomly allocated into three experimental groups: (1) control group; orthodontic tooth movement (OTM) of the upper left first molar with daily oral application of tap water (n = 20), (2) OTM with daily oral application of normal-dosed cetirizine (n = 19) and (3) OTM with daily oral application of high-dosed cetirizine (n = 19; Figure 1a) by oral gavaging. To reach a steady state of cetirizine in blood plasma [33], medication was started one week prior to the insertion of the orthodontic appliance and continued until the end of the experiment after 28 days of OTM. We established timing of medication and OTM in previous studies [25,27,32]. Following legal guidelines we euthanized rats at least 35 days after beginning of cetirizine treatment and 28 days after insertion of the orthodontic appliance by an intraperitoneal injection of 200 mg/kg Narcoren^®^ (Merial GmbH, Hallbergmoos, Germany). We performed three-dimensional cone-beam computed tomographies (CBCT) directly after insertion of the orthodontic appliance and after euthanasia. Furthermore, we performed micro-computed tomography (µCT) at the end of OTM, followed by paraffin histology and hematoxylin-eosin (HE) as well as tartrate-resistant acid phosphatase (TRAP) stainings.

### 2.3. Medication and Monitoring

Cetirizine (PubChem chemical ID number: 2678) was administered by the same investigator daily between 7:00 a.m. and 9:00 a.m. at the animal laboratory using oral gavage. Cetirizine tablets (10 mg, Ratiopharm GmbH, Ulm, Germany) were pulverized for application using a mixer mill (MM200, Retsch GmbH, Haan, Germany) and dosed at a precision of 0.1 mg (ABJ 120-4M, Kern & Sohn GmbH, Balingen, Germany). To ensure exact dosing oral gavage was chosen, which is an economical, convenient and relatively safe method of drug administration [34]. Pulverization and subsequent suspension of cetirizine is a permissible form of administration [35]. We calculated the clinical situation of oral intake using the formula by Reagan-Shaw et al. at a normal dose of 0.87 mg/kg in group (2) [36]. In group (3), we used a high dose of 3 mg/kg gross body weight, which was previously investigated by Meh et al. [17]. The corresponding dose of pulverized cetirizine (containing galenic components) for the experimental groups (2, 3) was vortexed in 1 mL tap water, while the control group (1) received 1 mL tap water alone. To monitor animal welfare, we recorded gross body weight (EBM2200-0, Kern & Sohn GmbH, Balingen, Germany) and adverse events daily.

### 2.4. Orthodontic Treatment

To induce orthodontic tooth movement a modified nickel-titanium closed coil tension spring (0.25 N, GAC Sentalloy^®^, Dentsply Sirona, Bensheim, Germany, 10-000-26) was inserted according to Kirschneck et al. under general anesthesia using a mixture of 6 mg xylazine and 90 mg ketamine per kg gross body weight, which was administered intraperitoneally [37]. Briefly, we inserted a coil spring connecting the first upper left molar (M1) and the upper incisors. The extended coil spring exerted a force of 0.25 N to M1 in an anterior direction. The right jaw side was left untreated and served as control. Lower incisors were regularly shortened once a week to prevent damage of the coil spring.

### 2.5. Preparation of Histological Slides

For histological analysis, we separated the left (treated) and right (untreated) sides of the jaw, demineralized them (10% Tris-buffered ethylene diamine tetra-acetic (EDTA) solution (pH 7.4)) for eight weeks and embedded them in paraffin. Paraffin blocks were cut with a rotating microtome (HM350, Microm International GmbH, Dreieich, Germany) in sagittal-oblique sections of 5 µm and fixed onto SuperFrost glass slides (SuperFrost Plus, Fisher Scientific GmbH, Schwerte, Germany).

### 2.6. TRAP (Tartrate-Resistant Acid Phosphatase) Staining

**Figure 2:**
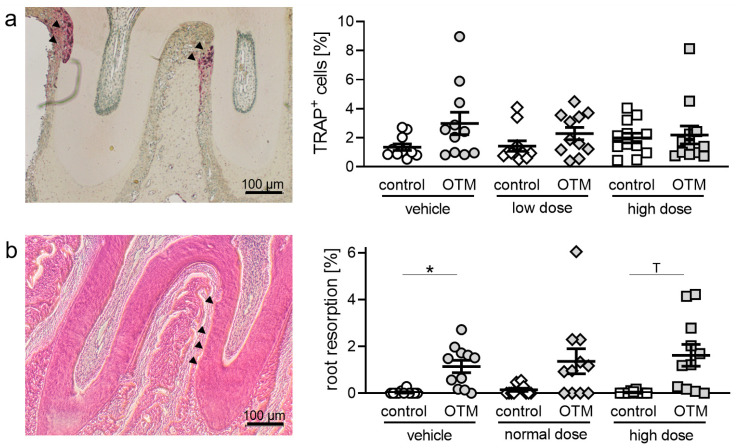
(**a**) Tartrate-resistant acid phosphatase (TRAP)^+^ (osteoclast-like) cells and (**b**) root resorption at the distobuccal root of the upper first molar at the control and orthodontically treated (OTM) jaw side after 28 days. n = 11. Statistics: Welch-corrected ANOVA followed by Games-Howell multiple comparison tests. ^T^ *p* < 0.1; * *p* < 0.05.

Sections were deparaffinized overnight at 37 °C and then hydrogenated by a descending alcohol series. TRAP buffer consisting of 1.64 g sodium acetate (6773.1, Carl Roth GmbH, Karlsruhe, Germany) and 23 g of di-sodium tartrate dihydrate (T110.1, Carl Roth GmbH., Karlsruhe, Germany) in 500 mL of H_2_O_dd_ (pH 5.0) was prepared and slides were incubated in this buffer for 10 min at room temperature. We freshly prepared a staining solution consisting of 40 mg of Naphthol AS-MX Phosphate Disodium Salt (N5000, Sigma-Aldrich, St. Louis, MO, USA), 4 mL of *N,N*-dimethylformamide (D4551, Sigma-Aldrich, St. Louis, MO, USA), 24 mg of Fast Red Violet LB Salt (F3381, Sigma-Aldrich, St. Louis, MO, USA), 2 mL Triton X-100 (T9284, Sigma-Aldrich, St. Louis, MO, USA) and 200 mL of the previously prepared TRAP buffer. After incubation for two hours at 37 °C in this staining solution, sections were rinsed in H_2_O_dd_ and counterstained with Mayer’s hematoxylin solution (51275, Sigma-Aldrich, St. Louis, MO, USA) for 3 min at room temperature and covered with Aquatex (1085620050, Merck KGaA, Darmstadt, Germany). Stained histological sections (Figure 2a) were digitized under the microscope (Olympus IX50 microscope in combination with DP2-SAL camera, Olympus, Hamburg, Germany). Evaluation of TRAP-positive area at the distobuccal root of the upper first molar in relation to the respective total root area within the same slice was carried out with the software ImageJ (Ver. 147, National Institutes of Health, Bethesda, MD, USA), as described in Kirschneck et al. [25,27,38].

### 2.7. Hematoxylin-Eosin (HE) Staining

Sections were deparaffinized at 60 °C for 30 min and transferred to xylene (9713.2, Carl Roth GmbH, Karlsruhe, Germany) for 20 min. They were hydrogenated by a descending alcohol series followed by staining with Mayer hematoxylin solution (1.07961.0500, Merck KG, Darmstadt, Germany) for 10 min. After incubation under running warm water for 5 min, slides were counterstained with eosin G solution 0.5% (X883.2, Carl Roth GmbH, Karlsruhe, Germany) for one minute. After rinsing the samples under warm tap water, they were dehydrated by an ascending series of alcohol. After incubation for 20 min in xylene, the coverslips were applied with entellan (1.07961.0500, Merck KGaA, Darmstadt, Germany). The stained histological sections (Figure 2b) were digitized under the microscope (Olympus IX50 microscope in combination with DP2-SAL camera, Olympus, Hamburg, Germany) and evaluation of the relative extent of root resorption area at the distobuccal root of the upper first molar in relation to the respective total root area within the same slice was performed with ImageJ (Ver.147, National Institutes of Health, Bethesda, MD, USA), as described in Kirschneck et al. [25,27,38].

### 2.8. Cranial Growth, Root Torque and Anterior Movement 

**Figure 3:**
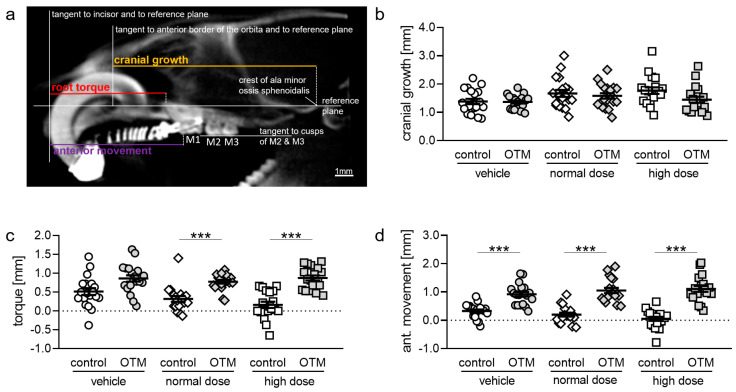
(**a**) Radiological cephalometry of cranial growth, root torque, and anterior movement of the first upper molar (M1) in reproducible two-dimensional sagittal cone-beam computed tomography (CBCT) planes of the rat skull based on the method described by Kirschneck et al. [32]. (**b**) Cranial growth, (**c**) root torque, and (**d**) anterior movement of M1 determined with CBCT. n ≥ 19. Statistics: (**b**,**c**) Welch-corrected ANOVA followed by Games–Howell multiple comparison tests. (**d**) Ordinary ANOVA followed by Bonferroni multiple comparison tests *** *p* < 0.001.

We performed cone-beam computed tomography (CBCT) according to Kirschneck et al. [37] (Veraviewepocs 3D R100/F40, Morita, Tokyo, Japan; 90 kV/5 mAs/9.4 s). Images were taken directly after coil spring insertion and euthanasia of the animals. Obtained CBCT images were analyzed by the blinded investigator for cranial growth and rate of OTM, as previously described [37] (Figure 3a). Before the measurements within the sagittal plane (Figure 3a) could be performed, a reference plane was identified. The nasal septum was parallelized to the X cursor line in the coronal plane (Y-slice). Next, the pulp centers of the ipsilateral incisor and the mesial root of the first upper molar were connected by the X cursor line in the transverse plane (Z-slice). We adopted the method of Kirschneck et al. [32] by using the Z cursor line in the sagittal plane (X-slice) to create an auxiliary tangent to the cusps of the second upper molar (M2) and the third upper molar (M3), which was then parallelly shifted through the crest of the ala minor of the sphenoid bone to create the reference plane (Figure 3a). According to Kirschneck et al. [37], cranial growth was calculated as the rise of distance from the tangent to the anterior border of the orbital cavity to the crest of the ala minor of the sphenoid bone. Calculation of OTM of the first upper left molar (M1) was based on anterior movement and root torque of M1. Cranial growth was calculated according to Kirschneck et al. [32]. Anterior movement of M1 was quantified as the decrease in distance from the mesial cusp tip of the first upper molar to the anterior incisor tangent, which was perpendicular to the reference plane [32]. Root torque was quantified as the decrease in distance from the mesial root tip of the first upper molar to the anterior incisor tangent [32]. To assess intra-rater reliability, we retested 20 randomly chosen CBCT data sets (at the start and after OTM, respectively) two weeks after the first measurements and calculated the concordance correlation coefficient according to Lin et al. [39], using MedCalc Statistical Software for Windows (ver. 19.1.7 MedCalc Software bv, Ostend, Belgium). Test-retest and interrater reliability of the CBCT procedure and quantification were previously shown by Kirschneck et al. [25,27].

### 2.9. Assessment of Distal Periodontal Bone Loss, Molar Inclination and Distance M1/M2 (µCT)

**Figure 4:**
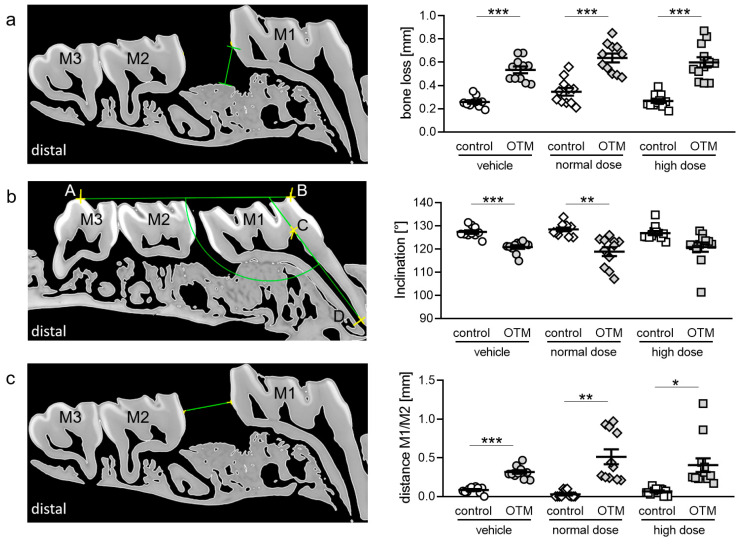
(**a**) Periodontal bone loss, (**b**) molar inclination (M1) and (**c**) distance between M1 and M2; measured using micro-computed tomography (µCT). n ≥ 10. Statistics: (**a**) Ordinary ANOVA followed by Bonferroni multiple comparison tests (**b**,**c**) Welch-corrected ANOVA followed by Games–Howell multiple comparison tests. * *p* < 0.05; ** *p* < 0.01; *** *p* < 0.001.

Micro-computed tomography (µCT; GE V-Tome-X S240, GE Healthcare, Chicago, IL, USA) was performed ex vivo using the Fast-Scan protocol (33 min, nanofocus tube, voxel size 4.5 µm; magnification 44.4 times, picture number 2000; timing 1000 ms; voltage 35 kV; electricity 145) at the Ostbayerische Technische Hochschule (OTH) Regensburg. After euthanasia and perfusion of the rats with 5% formaldehyde, we removed a segment of the upper rat jaw en-bloc, which consisted of the palate, alveolar bone and the three upper rat molars on both jaw sides. All connective tissue, oral mucosa, and the coil spring were carefully removed to reduce artefacts. The jaw segments were then preserved in a 5% formaldehyde solution overnight at 4 °C. The next day they were transferred to 0.1% formaldehyde for long-time storage. Three-dimensional images were analyzed using the image processing software VGSTUDIO MAX (Volume Graphics GmbH, Heidelberg, Germany) in cooperation with the OTH Regensburg. After blinding of the µCT data, we determined a reproducible plane as follows: On the jaw side of OTM within the transverse plane, the pulp centers of the mesial root of M1 and the distal root of M3 were connected using the cursor line. Within the sagittal sectional plane (Figure 4b), we located the plane that provided the full extent of the mesial root pulp of M1 while also showing the highest elevations of the distopalatal cusp of M3 (A) and the mesiopalatal cusp of M1 (B). Within this plane the mesial pulp horn (C) and the anterior wall of the mesial roots apical area (D) of M1 were located. Using the image processing software’s four-point goniometer and reference points, the angle of mesiodistal tipping of the first upper molar was measured with onward inclination being the orthodontically desired treatment effect representing OTM (Figure 4b). Periodontal bone loss around the first molar was determined distally as the distance between the enamel-cement border and the alveolar limbus on the treated and control sides (Figure 4a). To further asses OTM, the shortest distance between M1 and M2 was measured within the sagittal sectional plane using the image processing software’s caliper function (Figure 4c).

### 2.10. Statistical Methods

After testing for normal distribution (Shapiro–Wilk-test), either ordinary ANOVA followed by Bonferroni multiple comparison or Welch-corrected ANOVA followed by Games–Howell post-hoc tests was performed using GraphPad Prism version 8.4.3 for Windows, (GraphPad Software, San Diego, CA, USA). The significance level was set at *p* < 0.05. Symbols in figures (except Figure 1b) represent single data points, horizontal lines the arithmetic mean and vertical lines the standard error of the mean. Statistical analysis and exact *p*-values are presented as supplementary information.

## 3. Results

### 3.1. Time Flow Chart of Animal Experiment and Effects of Cetirizine and OTM on Animal Welfare

Male rats were randomly allocated into three experimental groups: (1) control group; orthodontic tooth movement (OTM) of the upper left first molar with daily application of tap water (n = 20) by oral gavage; (2) OTM with daily application of normal-dosed (0.87 mg/kg) cetirizine (n = 19); and (3) OTM with daily application of high-dosed (3 mg/kg) cetirizine by oral gavage (n = 19; Figure 1a). To reach steady state of cetirizine in blood plasma [33], medication was administered one week prior to the insertion of the orthodontic appliance and continued until the end of the experiment. We investigated possible side effects of cetirizine on animal welfare by daily monitoring gross body weight. All animals showed a continuous increase in gross body weight and survived until the last day of the experiment. The rats had a mean body weight of 170 ± 17 g starting medication, 196 ± 17 g at NiTi coil spring insertion and 258 ± 19 g after 28 days of OTM. Weight reduction on day seven was due to insertion of the NiTi coil spring. Neither normal nor high dose administration of cetirizine impacted on body weight compared to the control group (Table S1; Figure 1b).

### 3.2. Effects of Cetirizine and OTM on Osteoclastogenesis and Root Resorption.

First, we investigated osteoclastogenesis during OTM by performing tartrate-resistant acid phosphatase (TRAP) staining (Table S2, Figure 2a). After 28 days of tooth movement, we detected no significant changes in TRAP^+^ (osteoclast-like) cells in the control, normal, and high cetirizine dosage groups (Figure 2a). Cetirizine had no impact on osteoclastogenesis without and with OTM. We also tested for anabolic events of tooth movement and determined osteoblast numbers on the tension side of the alveolar bone (Table S3, Figure S1). We detected no effect of OTM or cetirizine medication on osteoblast number (Figure S1). Next, we determined root resorptions. We observed significantly increased root resorption at the OTM side of control animals (Table S4, Figure 2b). Cetirizine had no effect on root resorption at the control side and OTM side (Figure 2b).

### 3.3. Effects of Cetirizine and OTM on Cranial Growth, Root Torque and Anterior Movement.

We analyzed cone-beam computed tomography (CBCT) images (Figure 3a) and detected no effects of cetirizine medication on cranial growth on the control nor on the OTM side (Table S5, Figure 3b). We observed no effect of OTM on cranial growth in animals treated with tap water, normal dose or high dose cetirizine. Next, we analyzed the effects of OTM and cetirizine on orthodontic tooth movement, investigating root torque and anterior movement of M1 using CBCT. Root torque was increased with OTM in the vehicle group by tendency—that is not significantly (Table S6, Figure 3c)—while in the normal and high dose cetirizine groups we detected a significantly more root torque with OTM. Cetirizine medication had no effect on root torque on the control or the OTM side (Figure 3c). Anterior movement of M1 was significantly increased by OTM with tap water, normal and high dose cetirizine (Table S7, Figure 3d). Again, medication with cetirizine had no effect on M1 anterior movement on the control or the OTM side (Figure 3d).

### 3.4. Effects of Cetirizine and OTM on Periodontal Bone Loss, Molar Inclination and Anterior Movement.

To further assess possible effects of cetirizine, we measured periodontal bone loss distally of the upper first molar by analyzing µCT images (Table S8, Figure 4a). Orthodontic treatment led to significant periodontal bone loss in all groups with no effects of cetirizine (Figure 4a). Next, we measured desired onward inclination of M1 representing OTM (Table S9, Figure 4b). Onward inclination of M1 was significantly increased with OTM in the vehicle and the normal dose group, represented by the angular reduction measured, while we detected no significant effect of OTM on molar inclination in the high dose group (Figure 4b). Cetirizine medication affected M1 inclination only at a high dose. The extent of anterior tooth movement was analyzed by measuring the distance between M1 and M2 (Table S10, Figure 4c). Orthodontic treatment increased the distance in the vehicle, the low, and the high dose cetirizine group (Figure 4c). Medication with cetirizine showed no effect on the distance between M1 and M2 on the control or the OTM side (Figure 4c). 

## 4. Discussion

In our present study, we aimed to investigate, whether the H1-antihistamine cetirizine influences the rate of orthodontic tooth movement (OTM), cranial growth, root resorption, or periodontal bone loss, if administered at a clinically relevant dosage, but also a higher dosage, which has already been investigated in previous studies. We found that cetirizine had no significant effect on the rate of OTM, cranial growth, root resorption, periodontal bone loss or body weight development in both tested dosages. 

Two previous studies investigating the influence of cetirizine on OTM in male rats relied on digital calipers to measure the rate of OTM [17,18], while we used cone beam computed tomography (CBCT), micro-computed tomography (µCT) and software for our measurements, which allow more reliable and precise measurements [37]. OTM was quantified both as onward inclination, anterior movement and root torque of the first upper molar to address the different modalities of therapeutically induced tooth movement in orthodontics. The reason using both CBCT and µCT imaging is that only with CBCT imaging a longitudinal analysis was possible imaging the rats at the start and end of orthodontic treatment, thus, gaining the actual amount of tooth movement within each animal. The µCT analysis was performed to assess periodontal bone loss and used to corroborate findings on tooth movement by a second methodologically different analysis, which has the advantage of higher precision and resolution than CBCT imaging, but the disadvantage of only being able to record the endpoint after tooth movement, as radiation exposure does not allow in vivo measurements. µCT regarding M1 movement was thus used as an independent validation of CBCT longitudinal measurements of M1 movement. We chose to perform two-dimensional instead of three-dimensional measurements in both CBCT and µCT analyses, as these can be performed more reliably and reproducibly defining a two-dimensional measurement plane according to anatomical reference landmarks of the skull based on the native axial, coronal, and sagittal layers recorded by three-dimensional imaging [37]. Neither the CBCT nor the µCT analysis showed any significant effects of cetirizine on the investigated types of tooth movement. These results are in line with an earlier study by Kriznar et al. investigating OTM at a dosage of 10 mg/kg cetirizine daily per os (p.o.) in male Wistar rats. Even though a significant decrease of tooth movement occurred at day 7 in the cetirizine group compared to the control group, no significant differences in tooth movement were described at day 28 of OTM [18]. A later study by Meh et al., using similar methods at a dosage of 3 mg/kg cetirizine, showed no effect on day 7, but a significant decrease in tooth movement on day 28, 35, and 42 [17]. These findings are not in line with our results, which could be attributed to differences in bone metabolism of rats based on age. We used adolescent rats at 7 weeks of age, while Meh et al. used rats at weeks 13-14, which can be considered as young adulthood [40]. Our observation period was limited to 28 days, but this time frame successfully provided results in previous research [25,26,27,32,37,38]. In the life of an adolescent rat, 28 days correspond to 2.6 human years [40], which is considerably longer than the average duration of orthodontic treatment with fixed appliances of 19.9 months [41], and should therefore be adequate to simulate all stages of OTM. 

We investigated cetirizine at a dosage of 3 mg/kg as described by Meh et al., but also at a lower dosage calculated by using the formula by Reagan-Shaw et al., which enables dosage translation between animals and humans while considering body surface area [36]. Using the average European human body mass of 70.8 kg [42] and the clinically relevant daily dosage of 10 mg cetirizine p.o. in humans [43], we calculated a clinically relevant and corresponding dosage of 0.87 mg/kg in rats, which is considerably lower than the previously investigated dosages of 3 mg/kg [17] and 10 mg/kg [18].

Apart from our primary endpoint OTM, we also investigated cranial growth, root resorption and osteoclastogenesis to gain a more complete picture of the effects of cetirizine on bone metabolisms in the cranium and alveolar area in orthodontic context, as all these processes are clinically relevant in orthodontics (root resorptions are a frequent unwanted side effect of treatment, cranial growth modulation by orthopedic appliances is a frequently used therapy option) and linked at the cellular and bone level by the amount of osteoclastogenesis and osteoclast activity. 

Meh et al. showed that osteoclast volume density in the cetirizine group was significantly decreased relative to the appliance-only group [17]. Similarly Hwang et al. showed that TRAP-positive cell count was significantly lower in the cetirizine-injected group at day 28 of suture expansion [19]. On the contrary, we could see no significant effects of cetirizine on osteoclastogenesis using TRAP staining in any of the groups. We investigated periodontal bone loss using µCT images. No significant differences were shown between the control group and the two experimental groups. Furthermore, we investigated a possible influence of cetirizine on the extent of OIIRR, but could not see any effects of cetirizine between the groups.

So far there are no studies on the influence of cetirizine on cranial growth in rats, a recent study by Hwang et al. investigated the effects of cetirizine on bone remodeling after calvarial suture expansion in male rats [19]. The methodology of this study differs from our rat model and the investigated dosage of 10 mg/kg does not match our protocol, but the observation period of 28 days does. Intraperitoneal cetirizine injections led to a significantly higher bone volume density, bone/tissue volume percentage and narrower average suture width (µCT) as well as mineralized area and mineralized/fibrous area ratio (histomorphometry). Decreased bone resorption caused by the H1RA suppressing osteoclastic activity was suspected to be the underlying mechanism [19]. As cranial sutures play an important role in cranial growth [10], our results regarding sagittal cranial growth are comparable, but not in line with this study’s findings about the rate of suture expansion, which could be attributable to the higher dosage of cetirizine and different mode of drug administration. On the contrary, a study by Aasarød et al. showed that 3 mg/kg of cetirizine delivered by oral gavage over the course of 6 months had no effect on femur length, bone mineral density, and bone quality in female osteoporotic phenotype, as well as wild type mice [4], supporting our findings. A study by Folwarczna et al. [20] also showed comparable results in rapidly growing male rats. Loratadine, a second generation H1-antihistamine like cetirizine, did not affect the skeletal system significantly at a dose of 0.5 mg/kg and 5 mg/kg administered p.o. daily. The high dose of 50 mg/kg, however, significantly affected the longitudinal bone growth and cancellous bone mineralization, while no other effects on the skeletal system were observed. Due to a decreased body mass gain and increased liver mass, this effect might have been attributed to toxicity, and not only to loratadine’s effect on H1-receptors [20]. It seems that cetirizine and loratadine can affect bone growth and structure of the bone in higher dosages, while lower dosages seem to have no significant influence.

## 5. Conclusions

With the clinically relevant and the high dosage of cetirizine, we detected no relevant effects on tooth movement or any side effects of OTM. Cetirizine did not have a relevant impact on the rate of OTM, cranial growth, root resorption, or periodontal bone loss. This indicates that taking cetirizine in clinically relevant dosages during orthodontic treatment should have no undesired effects in humans in orthodontic context, a finding that needs to be corroborated in further clinical trials.

## Supplementary Materials

The following are available online at https://www.mdpi.com/2227-9059/8/12/583/s1, Figure S1: Osteoblast number at the surface of the alveolar bone in tension areas of the periodontal ligament in a standardized ROI of 15 µm × 160 µm. Table S1: Statistical analysis of body weight change during the experiment (F_W_ = 0.9213. df = 67.88. *p* = 0.4029) using Games–Howell’s multiple comparisons tests. Table S2: Statistical analysis of osteoblast number (F = 2.619; df = 56; *p* = 0.0338) using Bonferroni’s multiple comparisons tests. Table S3: Statistical analysis of TRAP^+^ (osteoclast-like) cells (F_W_ = 1.638; df = 28.27; *p* = 0.1824) using Games–Howell’s multiple comparisons tests. Table S4: Statistical analysis of root resorptions (F_W_ = 6.826; df = 25.88; *p* = 0.0004) using Games–Howell’s multiple comparisons tests. Table S5: Statistical analysis of cranial growth (F_W_ = 2.859; df = 50.04; *p* = 0.0240) using Games–Howell’s multiple comparisons tests. Table S6: Statistical analysis of root torque (F_W_ = 15.15; df = 50.93; *p* < 0.0001) using Games–Howell’s multiple comparisons tests. Table S7: Statistical analysis of anterior movement of M1 (F = 33.79; df = 110; *p* < 0.0001) using Bonferroni’s multiple comparisons tests. Table S8: Statistical analysis of periodontal bone loss (F = 28.89; df = 61; *p* < 0.0001) using Bonferroni’s multiple comparisons tests. Table S9: Statistical analysis of inclination (F_W_ = 13.99; df = 28.19; *p* < 0.0001) using Games–Howell’s multiple comparisons tests. Table S10: Statistical analysis of distance between M1/M2 (F_W_ = 28.03; df = 28.12; *p* < 0.0001) using Games–Howell’s multiple comparisons tests.
