# Supplementary material for: Effects of Histamine Receptor Antagonist Cetirizine on Orthodontic Tooth Movement
Source: Biomedicines. 2020 Dec 8;8(12):583. doi: 10.3390/biomedicines8120583 (PMC7762581; doi:10.3390/biomedicines8120583)
Supplement: Supplementary file 1 [file biomedicines-08-00583-s001.pdf]

Supplementary information

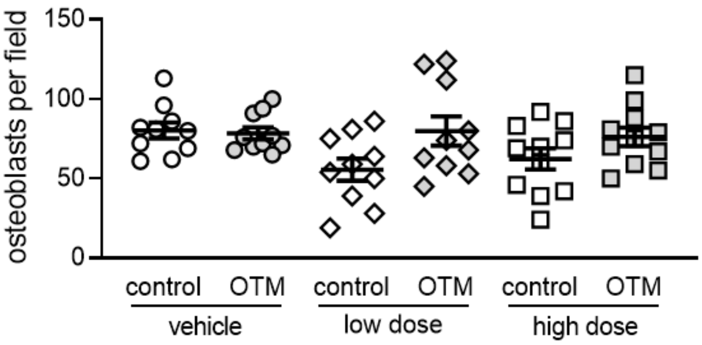

Figure S1: Osteoblast number at the surface of the alveolar bone in tension areas of the periodontal ligagment in a standardized ROI of 15µm x 160µm.

To determine osteoblast numbers on the alveolar bone at the tension side (Fig. S1), sections were stained with safranin-orange. To this aim, we incubated the slides 8 minutes in safranin solution (03982, Fluka, Charlotte, NC, USA), rinsed them in H_2O_d for 1 minute, followed by 10 minutes in picric acid (P6744, Sigma-Aldrich, St. Louis, MO, USA). After rinsing in H_2O_d for 20 sec, slides were incubated in a 0.1% light-green solution (115941, Sigma-Aldrich, St. Louis, MO, USA) with 0.1% acetic acid (3738.1 Carl Roth GmbH, Karlsruhe, Germany). After dehydration by an ascending series of alcohol, coverslips were applied with entellan and stained sections were digitized under the microscope (Olympus IX50 microscope in combination with a DP2-SAL camera, Olympus, Hamburg, Germany). Numbers of osteoblasts were counted at the surface of the alveolar bone in tension areas of the periodontal ligament in a standardized ROI of 15µm x 160µm in every slide.

Table S1: Statistical analysis of body weight change during the experiment ($F_W = 0.9213$. df = 67.88. $p = 0.4029$) using Games-Howell's multiple comparisons tests.

comparsion	95% CI of diff	p-value
control vs. normal dose	-17.77 to 12.26	0.8993
control vs. high dose	-8.828 to 20.27	0.6157
normal dose vs. high dose	-6.861 to 23.81	0.3869

Table S2: Statistical analysis of $TRAP^+$ (osteoclast-like) cells ($F_W = 1.638$. df = 28.27; $p = 0.1824$) using Games-Howell's multiple comparisons tests.

comparsion	95% CI of diff	p-value
vehicle: control vs. OTM	-4.334 to 1.074	0.3803
low dose control vs. OTM	-2.615 to 0.8980	0.6438
high dose control vs. OTM	-2.471 to 2.049	0.9996
vehicle control vs. low dose control	-1.424 to 1.280	>0.9999
vehicle control vs. high dose control	-1.907 to 0.6495	0.6354
low control vs. high dose control	-2.094 to 0.9806	0.8615
vehicle OTM vs. low dose OTM	-2.144 to 3.543	0.9642
vehicle OTM vs. high dose OTM	-2.322 to 3.904	0.9642
low dose OTM vs. high dose OTM	-2.289 to 2.471	>0.9999

Table S3: Statistical analysis of osteoblast number (F = 2.619. df = 56; p = 0.0338) using Bonferroni's multiple comparisons tests.

comparsion	95% CI of diff	p-value
vehicle: control vs. OTM	-25.02 to 28.62	>0.9999
low dose control vs. OTM	-51.22 to 2.423	0.1006
high dose control vs. OTM	-39.48 to 11.67	>0.9999
vehicle control vs. low dose control	-2.123 to 51.52	0.0925
vehicle control vs. high dose control	-8.279 to 44.13	0.4808
low control vs. high dose control	-32.98 to 19.43	>0.9999
vehicle OTM vs. low dose OTM	-28.32 to 25.32	>0.9999
vehicle OTM vs. high dose OTM	-23.99 to 28.42	>0.9999
low dose OTM vs. high dose OTM	-22.49 to 29.92	>0.9999

Table S4: Statistical analysis of root resorptions (F_W = 6.826. df = 25.88; p = 0.0004) using Games-Howell's multiple comparisons tests.

comparsion	95% CI of diff	p-value
vehicle: control vs. OTM	-2.034 to -0.1801	0.0178
low dose control vs. OTM	-3.076 to 0.6449	0.2918
high dose control vs. OTM	-3.205 to 0.03272	0.0556
vehicle control vs. low dose control	-0.3495 to 0.1193	0.5974
vehicle control vs. high dose control	-0.09888 to 0.1051	>0.9999
low control vs. high dose control	-0.1123 to 0.3487	0.5377
vehicle OTM vs. low dose OTM	-2.170 to 1.723	0.9988
vehicle OTM vs. high dose OTM	-2.208 to 1.255	0.9442
low dose OTM vs. high dose OTM	-2.487 to 1.981	0.9991

Table S5: Statistical analysis of cranial growth (F_W = 2.859. df = 50.04; p = 0.0240) using Games-Howell's multiple comparisons tests.

comparsion	95% CI of diff	p-value
vehicle: control vs. OTM	-0.3064 to 0.3424	>0.9999
low dose control vs. OTM	-0.3883 to 0.5609	0.9936
high dose control vs. OTM	-0.1513 to 0.7955	0.3368
vehicle control vs. low dose control	-0.7507 to 0.1830	0.4573
vehicle control vs. high dose control	-0.8316 to 0.06289	0.1267
low control vs. high dose control	-0.6126 to 0.4115	0.9910
vehicle OTM vs. low dose OTM	-0.5532 to 0.1221	0.3953
vehicle OTM vs. high dose OTM	-0.4446 to 0.2841	0.9833
low dose OTM vs. high dose OTM	-0.2957 to 0.5662	0.9319

Table S6: Statistical analysis of root torque (F_W = 15.15. df = 50.93; p < 0.0001) using Games-Howell's multiple comparisons tests.

comparsion	95% CI of diff	p-value
vehicle: control vs. OTM	-0.7163 to 0.01925	0.0720
low dose control vs. OTM	-0.7304 to -0.1812	0.0003
high dose control vs. OTM	-1.036 to -0.3892	<0.0001
vehicle control vs. low dose control	-0.1600 to 0.5502	0.5707
vehicle control vs. high dose control	-0.01614 to 0.7211	0.0678
low control vs. high dose control	-0.1812 to 0.4960	0.7274
vehicle OTM vs. low dose OTM	-0.2043 to 0.3799	0.9402
vehicle OTM vs. high dose OTM	-0.3339 to 0.3106	>0.9999
low dose OTM vs. high dose OTM	-0.3531 to 0.1542	0.8401

Table S7: Statistical analysis of mesialisation (F = 33.79. df = 110; p < 0.0001) using Bonferroni's multiple comparisons tests.

comparsion	95% CI of diff	p-value
vehicle: control vs. OTM	-0.9092 to -0.2768	<0.0001
low dose control vs. OTM	-1.177 to -0.5282	<0.0001
high dose control vs. OTM	-1.383 to -0.7340	<0.0001
vehicle control vs. low dose control	-0.1924 to 0.4483	>0.9999
vehicle control vs. high dose control	-0.04344 to 0.5973	0.1446
low control vs. high dose control	-0.1755 to 0.4734	>0.9999
vehicle OTM vs. low dose OTM	-0.4520 to 0.1887	>0.9999
vehicle OTM vs. high dose OTM	-0.5089 to 0.1319	0.8899
low dose OTM vs. high dose OTM	-0.3813 to 0.2676	>0.9999

Table S8: Statistical analysis of periodontal bone loss (F = 28.89. df = 61; p < 0.0001) using Bonferroni's multiple comparisons tests.

comparsion	95% CI of diff	p-value
vehicle: control vs. OTM	-0.4096 to -0.1437	<0.0001
low dose control vs. OTM	-0.4207 to -0.1611	<0.0001
high dose control vs. OTM	-0.4567 to -0.2083	<0.0001
vehicle control vs. low dose control	-0.2214 to 0.04451	0.5445
vehicle control vs. high dose control	-0.1391 to 0.1215	>0.9999
low control vs. high dose control	-0.04741 to 0.2067	0.6883
vehicle OTM vs. low dose OTM	-0.2325 to 0.02704	0.2373
vehicle OTM vs. high dose OTM	-0.1917 to 0.06233	>0.9999
low dose OTM vs. high dose OTM	-0.08900 to 0.1651	>0.9999

Table S9: Statistical analysis of inclination ($F_W = 13.99$. df = 28.19; $p < 0.0001$) using Games-Howell's multiple comparisons tests.

comparsion	95% CI of diff	p-value
vehicle: control vs. OTM	3.072 to 9.861	0.0001
low dose control vs. OTM	2.701 to 16.54	0.0050
high dose control vs. OTM	-1.053 to 13.09	0.1190
vehicle control vs. low dose control	-4.651 to 2.353	0.8996
vehicle control vs. high dose control	-3.149 to 4.132	0.9980
low control vs. high dose control	-2.064 to 5.344	0.7350
vehicle OTM vs. low dose OTM	-4.885 to 8.899	0.9208
vehicle OTM vs. high dose OTM	-6.951 to 7.036	>0.9999
low dose OTM vs. high dose OTM	-10.66 to 6.733	0.9790

Table S10: Statistical analysis of distance between M1/M2 ($F_W = 28.03$. df = 28.12; $p < 0.0001$) using Games-Howell's multiple comparisons tests.

comparsion	95% CI of diff	p-value
vehicle: control vs. OTM	-0.3158 to -0.1479	<0.0001
low dose control vs. OTM	-0.8214 to -0.1477	0.0050
high dose control vs. OTM	-0.6439 to -0.03277	0.0276
vehicle control vs. low dose control	-2.438e-005 to 0.1128	0.0501
vehicle control vs. high dose control	-0.03514 to 0.07074	0.8945
low control vs. high dose control	-0.09549 to 0.01836	0.3142
vehicle OTM vs. low dose OTM	-0.5349 to 0.1422	0.4116
vehicle OTM vs. high dose OTM	-0.3968 to 0.2194	0.9219
low dose OTM vs. high dose OTM	-0.3051 to 0.5204	0.9610
